# Gut microbiota composition in chemotherapy and targeted therapy of patients with metastatic colorectal cancer

**DOI:** 10.3389/fonc.2022.955313

**Published:** 2022-09-23

**Authors:** Yen-Cheng Chen, Chia-Hsien Chuang, Zhi-Feng Miao, Kwan-Ling Yip, Chung-Jung Liu, Ling-Hui Li, Deng-Chyang Wu, Tian−Lu Cheng, Chung-Yen Lin, Jaw-Yuan Wang

**Affiliations:** ^1^ Division of Colorectal Surgery, Department of Surgery, Kaohsiung Medical University Hospital, Kaohsiung Medical University, Kaohsiung, Taiwan; ^2^ Graduate Institute of Clinical Medicine, College of Medicine, Kaohsiung Medical University, Kaohsiung, Taiwan; ^3^ Institute of Information Science, Academia Sinica, Taipei, Taiwan; ^4^ Division of Gastroenterology, Department of Internal Medicine, Kaohsiung Medical University Hospital, Kaohsiung, Taiwan; ^5^ Institute of Biomedical Sciences, Academia Sinica, Taipei, Taiwan; ^6^ Department of Biomedical Science and Environmental Biology, Kaohsiung Medical University, Kaohsiung, Taiwan; ^7^ Department of Medicine, College of Medicine, Kaohsiung Medical University, Kaohsiung, Taiwan; ^8^ Graduate Institute of Medicine, College of Medicine, Kaohsiung Medical University, Kaohsiung, Taiwan; ^9^ Department of Surgery, Faculty of Medicine, College of Medicine, Kaohsiung Medical University Hospital, Kaohsiung Medical University, Kaohsiung, Taiwan; ^10^ Center for Cancer Research, Kaohsiung Medical University, Kaohsiung, Taiwan; ^11^ Pingtung Hospital, Ministry of Health and Welfare, Pingtung, Taiwan

**Keywords:** metastatic colorectal cancer, targeted therapy, *Lactobacillus* species, *Bifidobacterium* species, *Fusobacterium nucleatum*, *Klebsiella quasipneumoniae*

## Abstract

Studies have reported the effects of the gut microbiota on colorectal cancer (CRC) chemotherapy, but few studies have investigated the association between gut microbiota and targeted therapy. This study investigated the role of the gut microbiota in the treatment outcomes of patients with metastatic CRC (mCRC). We enrolled 110 patients with mCRC and treated them with standard cancer therapy. Stool samples were collected before administering a combination of chemotherapy and targeted therapy. Patients who had a progressive disease (PD) or partial response (PR) for at least 12 cycles of therapy were included in the study. We further divided these patients into anti-epidermal growth factor receptor (cetuximab) and anti-vascular endothelial growth factor (bevacizumab) subgroups. The gut microbiota of the PR group and bevacizumab-PR subgroup exhibited significantly higher α-diversity. The β-diversity of bacterial species significantly differed between the bevacizumab-PR and bevacizumab-PD groups (*P* = 0.029). *Klebsiella quasipneumoniae* exhibited the greatest fold change in abundance in the PD group than in the PR group. *Lactobacillus* and *Bifidobacterium* species exhibited higher abundance in the PD group. The abundance of *Fusobacterium nucleatum* was approximately 32 times higher in the PD group than in the PR group. A higher gut microbiota diversity was associated with more favorable treatment outcomes in the patients with mCRC. Bacterial species analysis of stool samples yielded heterogenous results. *K. quasipneumoniae* exhibited the greatest fold change in abundance among all bacterial species in the PD group. This result warrants further investigation especially in a Taiwanese population.

## Introduction

Colorectal cancer (CRC), a highly prevalent malignant disease globally, is the third most common cancer and the fourth leading cause of cancer-related deaths (1). In Asia, the incidence and mortality rates of CRC are the highest among all cancers, and the prevalence of CRC has been increasing in various countries including Japan, Korea, China, and Taiwan (2). In Taiwan, CRC has been the most common cancer since 2006 (3). Approximately 20%–25% of patients with CRC are initially diagnosed as having stage IV CRC with distant metastasis (4, 5). Although the overall survival (OS) of patients with CRC has increased, the treatment of metastatic CRC (mCRC) remains a clinical challenge. For example, patients with mCRC and *BRAF* mutation exhibited a poor response to systemic treatment and had an unfavorable prognosis (6, 7). The *RAS* gene mutation is a crucial factor for CRC tumorigenesis, invasion, and metastasis and can thus serve as a therapeutic agent (8). For mCRC treatment, doublet or triplet chemotherapy with fluoropyridine, oxaliplatin, or irinotecan is commonly used as a neoadjuvant therapy (9). Targeted therapy, including the use of an antivascular endothelial growth factor (VEGF) agent (e.g., bevacizumab ramucirumab, and aflibercept) and anti-epidermal growth factor receptor (EGFR) agents (e.g., cetuximab and panitumumab), has been suggested for mCRC treatment in combination with chemotherapy (5, 10–12). However, treatment outcomes have been unfavorable. The 3-year OS rate is approximately 50% (9), and less than 20% of patients survive beyond 5 years from the time of mCRC diagnosis (4).

State-of-the-art therapies for mCRC have been widely researched to improve treatment outcomes (13). The human gut microbiota plays a crucial role in human health and CRC treatment (14–16). The balance of the gut microbiota is essential for human health, and it affects the immune system, bowel health, and protection against pathogens. The dysregulation of the gut microbiota, or dysbiosis, is harmful and can result in cancer formation (15, 17). The toxicity of chemotherapeutic agents can alter the balance of the gut microbiota and break down the mucosal barrier of the bowel (18, 19). This mechanism induces gastrointestinal mucositis, thus affecting the quality of life of patients and leading to lower treatment compliance (19).

In CRC treatment, the gut microbiota is strongly associated with chemotherapy-related side effects (19, 20). For example, diarrhea is an adverse event (AE) that is commonly associated with irinotecan use, and SN-38 (the active metabolite of irinotecan) is the primary toxic agent. Some bacteria in the human bowel can secrete β-glucuronidase that converts SN-38G (the inactive metabolite) to SN-38, which may cause diarrhea. Silymarin, a bioflavonoid complex, can inhibit β-glucuronidase activity to alleviate diarrhea (21). Apart from side effects, the gut microbiota can also affect treatment outcomes. For instance, *Fusobacterium nucleatum* was demonstrated to promote the resistance of CRC cells to oxaliplatin through the activation of the autophagy pathway (22).

Some studies have reported an association between the gut microbiota and CRC chemotherapy (19, 23, 24); however, data regarding the association between the gut microbiota and targeted therapies for mCRC are lacking. In this study, we analyzed fecal samples from patients with mCRC to identify potential candidate bacteria related to treatment outcomes and to AEs that are associated with combination chemotherapy and targeted therapy. The findings of this study can help elucidate the role of the gut microbiota in targeted therapies for CRC. The present study was approved by the Institutional Ethics Committee of our hospital (KMUHIRB-E(II)-20180012).

## Material and methods

### Patients

We enrolled 110 patients with mCRC between July 2017 and June 2018. Abdominal computed tomography (CT), colonoscopy, and histopathology were used to establish the definite diagnosis of mCRC. A multidisciplinary panel consisting of colorectal surgeons, gastroenterologists, medical oncologists, radiologists, radiation oncologists, and pathologists discussed the treatment program. We first collected stool samples before the initiation of chemotherapy or targeted therapy. Chemotherapy with FOLFIRI (folinic acid, fluorouracil [5-FU], and irinotecan) was administered to all the patients. According to the patients’ general condition and nutritional status as well as the presence of metastasis, comorbidities, and *RAS* gene variations, we selected targeted therapy involving the use of either an anti-VEGF agent (bevacizumab [Avastin, Roche, Basel, Switzerland]) or an anti-EGFR agent (cetuximab [Erbitux, Merck, Darmstadt, Germany]) as the first-line treatment. Bevacizumab can be used for patients with either the wild-type or *RAS* gene variants, whereas cetuximab can only be used for patients with the wild-type *RAS* gene because of the drug’s mechanism of action (25, 26). The treatment regimen comprised a 120-min intravenous (IV) infusion of cetuximab (500 mg/m^2^) or bevacizumab (5 mg/kg) on day 1, followed by a 4-h IV infusion of irinotecan (180 mg/m^2^) plus normal saline (500 mL) and then a 42–46-h IV infusion of leucovorin (200 mg/m^2^), 5-FU (2800 mg/m^2^), and normal saline (500 mL). This regimen was repeated once every 2 weeks. During this period, we measured the serum carcinoembryonic antigen (CEA) level throughout each chemotherapy cycle.

**Figure 1:**
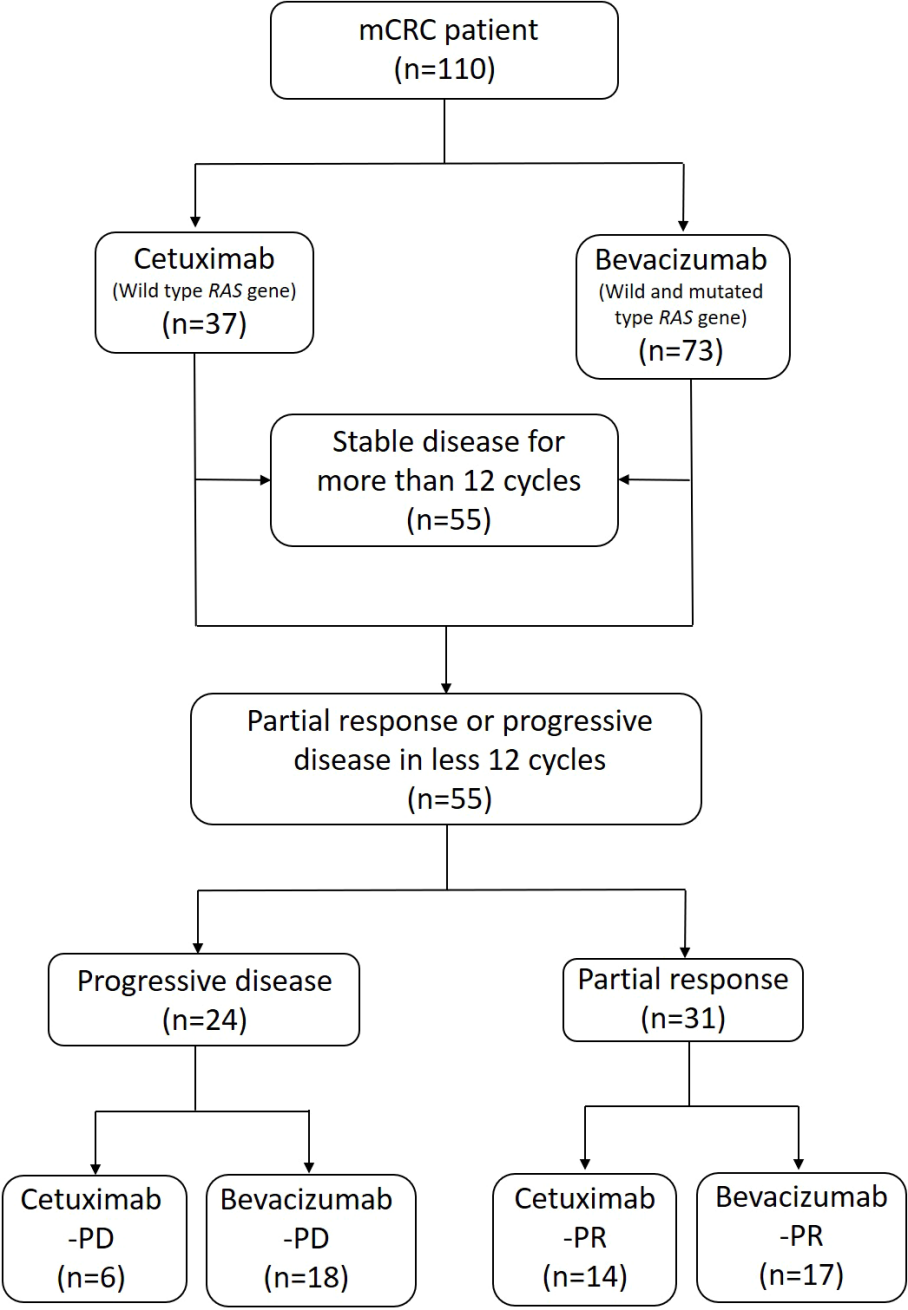
Flowchart of patient selection and classification. Anti-EGFR (cetuximab) and anti-VEGF (bevacizumab) agents were administered in accordance with the patients’ general condition and with results on *RAS* gene expression. The patients received standard treatment for mCRC in combination with a FOLFIRI (folinic acid, 5-FU, and irinotecan) chemotherapy regimen. According to the patients’ clinical response, we classified them into “progressive disease” and “partial response” groups. The patients were further classified into four subgroups with cetuximab-PD, bevacizumab-PD, cetuximab-PR, and bevacizumab-PR for the subsequent stool analysis.

We performed enhanced abdominal CT or magnetic resonance imaging (MRI) after every six cycles of therapy or if the patients were determined to have abnormal serum CEA levels during two consecutive tests. Response measurements were based on the Response Evaluation Criteria in Solid Tumors (RECIST), version 1.1 (27, 28); partial response (PR), stable disease (SD), and progressive disease (PD) were evaluated on the basis of CT or MRI findings. The patients who exhibited a PR or PD during at least 12 cycles of therapy were included in the study. Those who exhibited a SD during 12 or more cycles of therapy were excluded. We divided the patients into the PD and PR groups according to their clinical response. For further stool analysis, the patients were divided into four subgroups according to the first-line targeted therapy they received: cetuximab-PD, bevacizumab-PD, cetuximab-PR, and bevacizumab-PR. The patient selection and classification processes are presented in **Figure 1**.

During each cycle of neoadjuvant and adjuvant chemotherapy, AEs were examined using the Common Terminology Criteria for Adverse Events, version 4.0 (29). In the substantial diarrhea group, we included patients with grade 3 or grade 2 diarrhea and those who had grade 1 diarrhea at least five times during the treatment course. The median number of treatment cycles was 10 (range: 6 to 20). In the minor or no diarrhea group, we included the patients who did not experience the aforementioned diarrhea episodes.

### Metagenomics DNA extraction

All stool samples were collected in our ward during hospitalization with the assistance of a nurse. We used an Fe-Col fecal collection device (Alpha Laboratories, Eastleigh, United Kingdom) to collect the stool sample and prevent contamination. The samples were temporarily stored in a −4°C freezer and then transferred into a −80°C freezer within 6 h. We used the QIAamp Fast DNA Stool Mini Kit (Qiagen, Hilden, Germany) to extract high-quality genomic DNA (gDNA) from frozen stool samples. DNA extraction was performed in accordance with a published protocol (30). The quantity and quality of the extracted gDNA were measured using an ND-1000 spectrophotometer (Nanodrop Technology, Wilmington, USA), Qubit 4 Fluorometer (Thermo Fisher, Waltham, USA), and Agilent 4200 TapeStation System (Agilent Technologies, Santa Clara, USA); these measurements included the concentration, purity, and integrity of gDNA. The protocol that yielded a higher quantity and quality of gDNA was then used to extract the gDNA of the remaining samples. The gDNA samples were stored at −80°C until library preparation and sequencing.

### Library preparation and sequencing (Illumina)

Sequencing libraries were prepared using the Nextera DNA Flex kit (Illumina) in accordance with the manufacturer’s instructions. One paired-end library with an insert size of approximately 320 bp was constructed for each sample. Libraries were normalized by performing the Qubit assay; the pooled library was then sequenced on one lane on a NovaSeq6000 platform (NovaSeq Control Software 1.6.0/RTA v3.4.4) with a 2 × 150 setup by using the NovaSeqXp workflow in the S1 mode flow cell. The Bcl to FastQ conversion was performed using bcl2fastq_v2.20.0.422 in CASAVA genetic analysis software. The quality scale used was Sanger/phred33/Illumina 1.8 +.

#### Library construction

For library construction, we extracted DNA from stool samples. After performing quality control, we used the qualified samples for library construction. The sequencing library was constructed through the random fragmentation of the DNA sample, followed by 5′ and 3′ adapter ligation. Moreover, we used tagmentation to combine fragmentation and ligation reactions into a single step, thus considerably increasing the efficiency of the library construction process. Adapter-ligated fragments were amplified by performing the polymerase chain reaction and then purified on a gel.

#### Sequencing

For cluster generation, the library was loaded into a flow cell where fragments were captured on a lawn of surface-bound oligos that were complementary to library adapters. Each fragment was amplified into distinct clonal clusters through bridge amplification. When cluster generation was completed, the templates were ready for sequencing.

### Quality control and taxonomic classification

The quality control of sequencing data performed using KneadData v.0.7.10 (https://huttenhower.sph.harvard.edu/kneaddata/) included read quality checks, base quality trimming, and human genome decontamination (hg37dec_v0.1). The taxonomic assignment was conducted using Kraken2 v.2.1.2 (31) on the basis of the alignment of minimizers from *k*-mers against the complete genome database in RefSeq for archaea, bacteria, fungi, and viruses. In addition, we added the unexplored human microbiome genome from another study (32) to enhance the sensitivity for taxonomic classification. After classification, the abundance levels of taxa were re-estimated using Bracken v.2.2 (https://ccb.jhu.edu/software/bracken/) at the species level (33).

### Diversity analysis and differential taxa identification

We examined the β-diversity of the samples in different subgroups by using QIIME2 v.2020.2.0 (https://qiime2.org) (34). The classification report from Bracken was converted using QIIME2, and the α-diversity (Shannon index) and β-diversity (Bray–Curtis) were calculated. The diversity results were visualized through boxplots and principal co-ordinate analysis plots in R software. We performed the Mann–Whitney *U* test to analyze differences in the α-diversity index and performed a permutational multivariate analysis of variance by using 999 iterations to examine differences in Bray–Curtis matrices between the groups. Statistical test results with *P* < 0.05 were considered significant.

The differential abundance analysis was performed using DESeq2 package v.1.30.1 (35). Our hypothesis was tested using the Wald test, and *P* values were adjusted using the Benjamini–Hochberg method. Taxa that met our statistical criteria (adjusted *P* < 0.05, >2 shrunken log_2_ fold changes, and >1000 base means) were considered differentially abundant between the two patient groups.

### Statistical analysis

All statistical analyses were performed using SPSS version 20 (IBM, Armonk, NY, USA). The chi-square test was performed to compare categorical data. *P* < 0.05 indicated statistical significance. Descriptive statistics are presented as proportions and means.

## Results

### Clinical response

The characteristics of the patients with mCRC at diagnosis and their gene variation profiles are presented in **Table 1**. Of the 110 patients with mCRC, 55 had a SD after more than 12 cycles of chemotherapy and were subsequently excluded from this study. The other 55 patients met the inclusion criteria of having a clear PD or PR. The PD and PR groups included 24 and 31 patients, respectively. Six patients exhibited a PD after receiving cetuximab and were included into the cetuximab-PD subgroup. Similarly, 18 patients were included into the bevacizumab-PD subgroup. The cetuximab-PR and bevacizumab-PR subgroups consisted of 14 and 17 patients, respectively. No patient presented a complete response after treatment.

**Table 1:** Patient Characteristics at Diagnosis and Gene Variation Profiles (n = 55).

Characteristic	
**Age (years, median) (range)**	62 (38-88)
**Gender**	
Male	26 (47.3%)
Female	29 (52.7%)
**BMI kg/m^2^ (mean) (range)^b^**	24.1 (18.7-34.8)
**Location**	
Cecum	3 (5.5%)
Ascending colon	7 (12.7%)
Transverse colon	6 (10.9%)
Descending colon	5 (9.1%)
Sigmoid colon	15 (27.3%)
Rectosigmoid junction	10 (18.2%)
Rectum	9 (16.4%)
**Sidedness**	
Right colon	16 (29.1%)
Left colon	39 (70.9%)
**Clinical stage**	
IVA	33 (60.0%)
IVB	13 (23.6%)
IVC	9 (16.4%)
***KRAS* mutation**	21/55 (38.2%)
***NRAS* mutation**	4/55 (7.3%)
***BRAF* mutation**	3/55 (5.5%)
**Ileostomy/colostomy prior to therapy**	
Yes	11 (20.0%)
No	44 (80.0%)
**Radiotherapy**	
Yes	38 (69.1%)
No	17 (30.9%)

Fourteen patients experienced substantial diarrhea during the treatment course. The number of patients with substantial diarrhea was equal in both the cetuximab (7 patients, 35%) and bevacizumab (7 patients, 20%) groups. We observed no significant difference in the clinical response or diarrhea frequency between the cetuximab and bevacizumab groups (**Table 2**).

**Table 2:** Results for patients by clinical response and diarrhea type.

	Cetuximab (N=20)	Bevacizumab (N=35)	*P* value
Clinical Response Progressive Disease Partial Response	6 (30%) 14 (60%)	18 (51.4%) 17 (48.5%)	0.721
Diarrhea Substantial Minor or no	7 (35%) 13 (65%)	7 (20%) 28 (80%)	0.279

### α-Diversity

**Figure 2:**
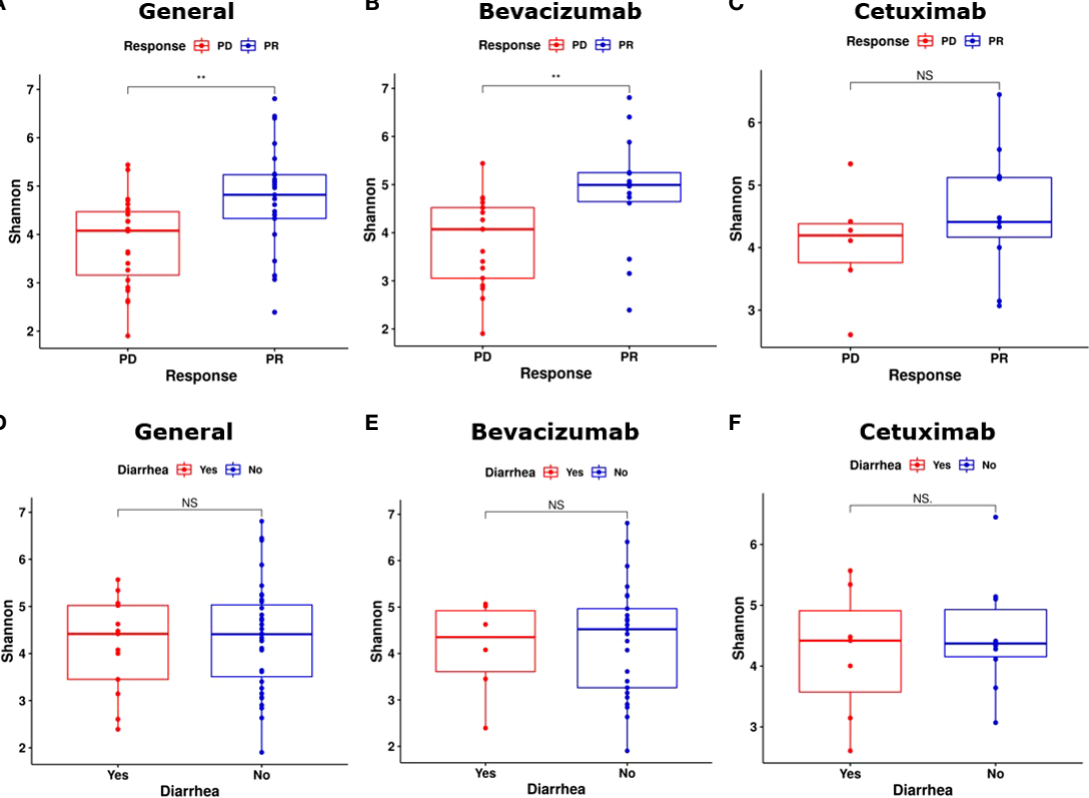
Results on α-diversity of groups and subgroups. **(A)** The PR group had a significantly higher α-diversity than did the PD group (*P* < 0.01). **(B)** α-diversity significantly differed between the bevacizumab-PD and PR subgroups (*P* < 0.01). **(C)** Bacterial species richness did not significantly differ between the cetuximab-PR and cetuximab-PD subgroups (*P* = 0.35). **(D)** α-diversity did not significantly differ between the “substantial diarrhea” and “minor or no diarrhea” groups (*P* = 0.57). **(E, F)** α-diversity did not significantly differ between the bevacizumab and cetuximab diarrhea subgroups (bevacizumab-PR vs. bevacizumab-PD and cetuximab-PR vs. cetuximab-PD, *P* = 0.72 and *P* = 1.00, respectively). NS, Not significant; **: *P* < 0.01.

α-Diversity was calculated to compare the diversity and richness of bacterial species among the groups and subgroups (36). The gut microbiota of the PR group exhibited significantly higher α-diversity than that of the PD group (*P* < 0.01, **Figure 2A**). The gut microbiota of the bevacizumab-PR subgroup demonstrated significantly higher α-diversity than that of the bevacizumab-PD subgroup (*P* < 0.01, **Figure 2B**). The α-diversity of the gut microbiota did not significantly differ between the cetuximab-PD and cetuximab-PD subgroups (*P* = 0.35, **Figure 2C**).

We compared the α-diversity of the substantial diarrhea and minor or no diarrhea groups. The gut microbiota of the minor or no diarrhea group exhibited nonsignificant α-diversity (*P* = 0.57, **Figure 2D**). No significant differences in the α-diversity of the gut microbiota were noted between the patients who developed substantial diarrhea in the bevacizumab subgroups and those in the cetuximab subgroups (bevacizumab-PR vs. bevacizumab-PD and cetuximab-PR vs. cetuximab-PD, *P* = 0.72 and *P* = 1.00, **Figures 2E, F**, respectively).

### β-Diversity

**Figure 3:**
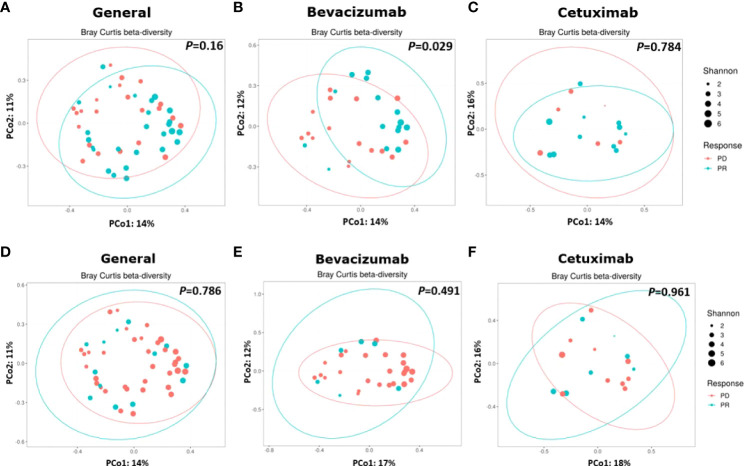
Results on β-diversity of treatment groups and subgroups. **(A)** β-diversity did not significantly differ between the PD and PR groups (*P* = 0.16); **(B)** Bacterial species significantly different between the bevacizumab-PD and PR subgroups (*P* = 0.029). **(C)** β-Diversity did not significantly differ between the cetuximab-PD and PR subgroups (*P* = 0.784). **(D)** β-diversity did not significantly differ between the substantial diarrhea and minor or no diarrhea groups (*P* = 0.786). **(E, F)** β-diversity did not significantly differ between the bevacizumab (*P* = 0.491) and cetuximab (*P* = 0.961) subgroups.

β-Diversity was calculated to compare the compositional differences of bacterial species among the groups and subgroups (37). The β-diversity of the gut microbiota did not significantly differ between the PD and PR groups (*P* = 0.16, **Figure 3A**). However, the β-diversity of bacterial species in the bevacizumab-PR subgroup significantly differed from that of the bevacizumab-PD subgroup (*P* = 0.029, **Figure 3B**). The β-diversity of the gut microbiota did not significantly differ between the cetuximab-PD and cetuximab-PR subgroups (*P* = 0.784, **Figure 3C**).

No significant difference in the β-diversity of the gut microbiota (*P* = 0.786, **Figure 3D**) was noted between the substantial diarrhea and minor or no diarrhea groups. Moreover, no significant difference in the β-diversity of the gut microbiota was noted in the patients with substantial diarrhea and those with minor or no diarrhea between the bevacizumab (*P* = 0.491, **Figure 3E**) and cetuximab (*P* = 0.961, **Figure 3F**) subgroups.

### Taxon abundance

**Figure 4:**
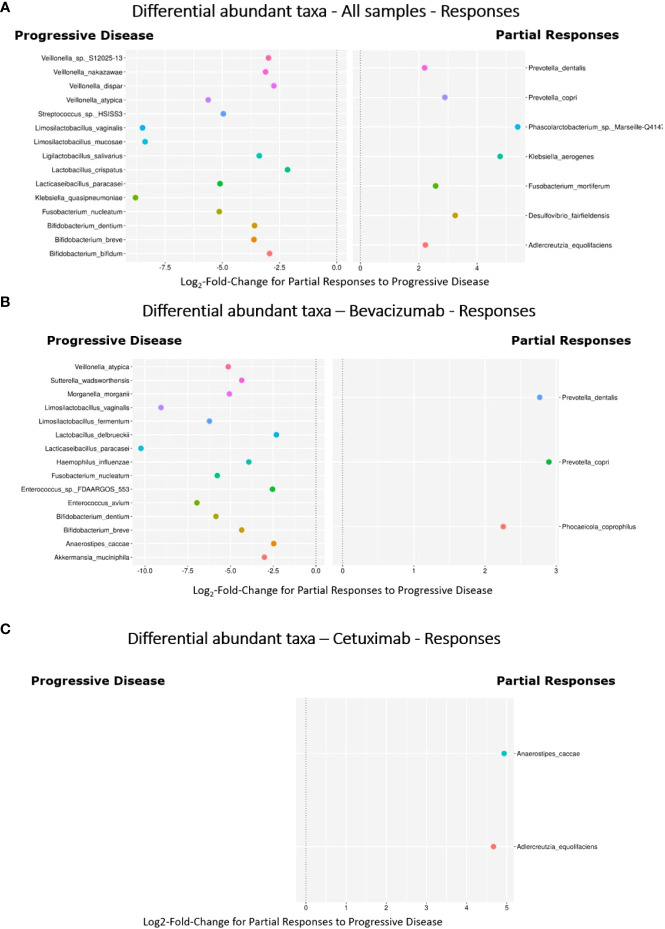
Results of the log_2_ fold change in the disease progression and partial response groups and subgroups of bevacizumab and cetuximab. Each dot indicates a specific bacteria species with a significant difference (*P* < 0.05) indicated on the *y*-axis. Positive values on the *x*-axis indicate that the bacterial species was significantly more abundant in the PR than in the PD group and subgroups. Negative values indicate the opposite. The zero point indicates the equal fold of abundance in both the groups (2^0^). Only log_2_ fold change values >|2| are shown. **(A)** Bacterial expression was compared between the PD and PR groups. *Fusobacterium nucleatum* in the PD group was located at approximately the −5 point (log_2_ fold change = −5.13, *P_adj_* = 3.37e-6), indicating that the expression of *F. nucleatum* was higher in the PD group than in the PR group by approximately 32 (2^5^) times. *Prevotella copri* in the PR group was located at approximately 3 point (log_2_ fold change = 2.90, *P_adj_* = 0.043), indicating that the expression of *P. copri* was higher in the PR group than in the PD group by approximately 8 (2^3^) times. **(B)** In the bevacizumab-PD subgroup, *Lacticaseibacillus paracasei* exhibited the highest fold change in abundance by more than 1000 (2^10^) times compared with the bevacizumab-PR subgroup (log_2_ fold change = −10.23, *P_adj_* = 8.28e-8). Other *Lactobacillus* species, including *Limosilactobacillus vaginalis*, *Limosilactobacillus fermentum*, and *Lactobacillus delbrueckii*, exhibited a higher fold change in abundance in the PD group than in the PR subgroup (log_2_ fold change = −9.06, *P_adj_* = 4.15e-10; log_2_ fold change = −6.22, *P_adj_* = 8.90e-6; and log_2_ fold change = −2.32, *P_adj_* = 6.22e-6, respectively). *Bifidobacterium dentium* and *Bifidobacterium breve* presented a higher fold of abundance (log_2_ fold change = −5.85, *P_adj_* = 8.90e-6 and log_2_ fold change = −4.34, *P_adj_* = 4.66e-5, respectively). *F. nucleatum* exhibited a higher fold change in abundance by approximately 64 (2^6^) times (log_2_ fold change = −5.77, *P_adj_* = 2.84e-4). In the bevacizumab-PR subgroup, *P. dentalis* and *P. copri* revealed a higher fold change in abundance than did the bevacizumab-PD subgroup by approximately 8 (2^3^) times (log_2_ fold change = 2.77, *P_adj_* = 4.41e-3 and log_2_ fold change = 2.90, *P_adj_* = 7.58e-3, respectively). **(C)** In the cetuximab-PD subgroup, no bacterial species presented a significantly different fold change in abundance. *Anaerostipes caccae* (log_2_ fold change = 4.94; *P_adj_* = 2.47e-3) and *Adlercrentzia equolifaciens* (log_2_ fold change = 4.70; *P_adj_* = 6.92e-3) revealed a higher fold change in abundance in the cetuximab-PR subgroup than in the cetuximab-PD subgroup.

To analyze bacterial taxa, we compared bacterial species expression among the groups and subgroups. Bacterial species that were not significantly differentially abundant after treatment were screened out, and differences in abundance are expressed as log_2_ fold changes. **Figure 4A** illustrates the differential taxon abundance of the PD and PR groups. *K. quasipneumoniae* in the PD group had a greater fold change in abundance than did *K*. *quasipneumoniae* in the PR group, and the difference between them in the fold change in abundance was the greatest among all bacterial species in the PD group. *Limosilactobacillus mucosae* exhibited the second greatest difference in the fold change in abundance between the PD (the greater of the two) and PR groups. *Veillonella atypica*, *Veillonella dispar*, *Veillonella nakazawae*, and *Veillonella* S12025-13 in the PD group demonstrated greater fold changes in abundance than in the PR group. The *Lactobacillus* species had a higher abundance in the PD group than in the PR group. *Bifidobacterium dentium*, *Bifidobacterium breve*, and *Bifidobacterium bifidum* had higher abundance in the PR group than in the PD group. The abundance of *F. nucleatum* in the PD group was approximately 32 (2^5^) times higher than that in the PR group.

*Phascolarctobacterium* sp. *Marseille-*Q4147 in the PR group exhibited a greater fold change in abundance than in the PD group, and the difference between them in the fold change in abundance was the greatest among all bacterial species in the PR group. Similarly, *Prevotella dentalis* and *Prevotella copri* in the PR group exhibited greater fold changes in abundance than in the PD group.

In the bevacizumab-PD subgroup, *Lacticaseibacillus paracasei*, *Li*. *Vaginalis*, *Limosilactobacillus fermentum*, and *Lactobacillus delbrueckii* exhibited greater fold changes in abundance than in the bevacizumab-PR subgroup. *Lacti paracasei* demonstrated the largest fold change in abundance (>1000 [2^10^] times) in the bevacizumab-PD subgroup. *B. dentium* and *B. breve* in the bevacizumab-PD subgroup presented greater fold changes in abundance than in the bevacizumab-PR subgroup. The fold change in the abundance of *F. nucleatum* in the bevacizumab-PD subgroup was nearly 64 (2^6^) times greater than that of *F. nucleatum* in the bevacizumab-PR subgroup. The fold changes in the abundance of *P. dentalis* and *P.* copri in the bevacizumab-PR subgroup were approximately 8 (2^3^) times greater than those of their counterparts in the bevacizumab-PD subgroup (**Figure 4B**).

In the cetuximab-PD subgroup, no bacterial species were significantly differentially abundant. *Anaerostipes caccae* and *Adlercrentzia equolifaciens* demonstrated greater fold changes in abundance in the cetuximab-PR subgroup than in the cetuximab-PD subgroup (**Figure 4C**).

## Discussion

The human gut microbiota is closely associated with CRC development (15, 18, 38, 39). In healthy individuals, the diversity and balance of the intestinal microbiota are essential to maintain the mucosal barrier and prevent pathogen invasion (15, 18, 38, 39). A balanced gut microbiota combined with favorable genetic or immune conditions, lifestyle, diet, and environmental factors contribute to intestinal homeostasis. The dysregulation of these factors can alter the gut microbiota and lead to gut dysbiosis (18, 38, 39). In the present study, the stool samples of the PR group and the bevacizumab-PR subgroup demonstrated significantly higher α-diversity of bacterial species than those of the PD group and the bevacizumab-PD subgroup, respectively. These results indicate that higher intestinal bacterial diversity may be helpful in the treatment of mCRC.

The α-diversity of bacterial species in the stool samples of the patients who received cetuximab did not significantly differ between the PR and PD groups. This finding may be attributable to 1) the limited number of the patients included in this study or 2) selection bias caused by the administration of cetuximab to only patients with the wild-type *RAS* gene. The *KRAS* gene, a member of the *RAS* gene family, is associated with many signaling pathways, including those related to inflammation, the immune system, and metabolism. The gut microbiota is a key factor because it maintains intestinal homeostasis through these pathways (40). Variations in the *KRAS* gene can disrupt normal signaling functions and hamper intestinal homeostasis. The accumulative effect can result in CRC tumorigenesis and affect CRC treatment outcomes (40). Microbiota can significantly differ between CRC tissues with and without *KRAS* gene variations (41). In clinical practice, anti-EGFR agents (e.g., cetuximab) are unsuitable for treating patients with *KRAS* gene variations (25, 26). In our study, those who received cetuximab were all wild-type *KRAS* gene carriers, whereas the patients who received bevacizumab comprised both wild-type and *KRAS* gene variation carriers (**Figure 1**). This selection bias may have affected the α-diversity results. However, more evidence is necessary to determine the relationship between intestinal bacterial diversity and *KRAS* gene variations.

We observed no significant difference in the β-diversity of bacterial species between the PD and PR groups. This result indicates the presence of similar intestinal bacterial composition in the PR and PD groups. The human gut microbiota can be individualized under the effect of both genetic and environmental factors (42–44). Dietary habit is an especially crucial environmental factor that can modulate microbiota composition (42, 44). Most of our included patients lived in southern Taiwan and shared similar food, culture, and climate. This may partially explain our β-diversity results, which indicated that the treatment outcome did not depend only on the gut microbiota. Age, sex, genetic factors, and nutritional status also affect CRC treatment outcomes. β-diversity was significantly different between the bevacizumab-PD and bevacizumab-PR subgroups. This result suggests that the composition of the gut microbiota is associated with the treatment response in specific subgroups. During our treatment course, the bevacizumab group comprised both the wild-type and *RAS* gene variation carriers (**Figure 1**). The *KRAS* gene is associated with various signaling pathways and can alter treatment outcomes (26, 40, 41). The relationship between the *KRAS* gene and human gut microbiota remains unclear. More studies, such as those investigating orthotopic rectal cancer by using animal models, should be conducted to elucidate this relationship in the future (16). Additional stool samples from the patients in the cetuximab-PD and cetuximab-PR subgroups may be necessary to obtain more compelling results.

In our experience, patients with mCRC typically require longer courses of systemic therapy. In this situation, oxaliplatin-based chemotherapy, such as FOLFOX (folinic acid, 5-FU, and oxaliplatin) may cause severe or irreversible peripheral neuropathy. Therefore, we used a FOLFIRI regimen as the initial therapy for mCRC. In this study, all our patients received chemotherapy with the FOLFIRI regimen, and the most frequent AE of the FOLFIRI regimen is diarrhea. The human gut microbiota plays a role in mitigating diarrhea caused by chemotherapy (45–47). Antibiotic-associated diarrhea caused by *Clostridium difficile* is a well-known condition that is the result of gut dysbiosis (45, 48). Normal intestinal commensal bacteria can inhibit pathogen growth and prevent pathogen-induced diarrhea (45). Many studies have reported that probiotics, including *Bifidobacterium*, *Lactobacillus*, and yeast, can improve gut microbiota balance and inhibit pathogen colonization. Through this mechanism, probiotics can prevent or treat pathogen-induced diarrhea (45, 47). Bacteria that produce β-glucuronidase can aggravate diarrhea (21, 23, 24), and β-glucuronidase inhibitors can be used for the symptomatic relief of diarrhea (21, 49). In theory, the elimination of bacteria that produce β-glucuronidase can also alleviate diarrhea. However, we failed to identify relevant bacterial species in our stool sample analysis.

DESeq2 analysis is a powerful parametric approach to compare targeted metagenomics (50, 51).We observed heterogeneous results when comparing the PD and PR groups. The *Bifidobacterium* and *Lactobacillus* species all exhibited significantly greater fold changes in abundance in the PD group than in the PR group. Moreover, the *Bifidobacterium* and *Lactobacillus* species demonstrated greater fold changes in abundance in the bevacizumab-PD subgroup than in the bevacizumab-PR subgroup. *Bifidobacterium* and *Lactobacillus* species are widely used in commercial probiotic supplements (52–54). Some studies have reported an association between *Bifidobacterium* species and CRC tumorigenesis (47). The effects of *Bifidobacterium* and *Lactobacillus* species on CRC chemotherapy and immune therapy outcomes have been studied (47, 55). However, how *Bifidobacterium* and *Lactobacillus* species affect the outcomes of targeted therapies for mCRC is not yet clearly understood.

*Bifidobacterium* species can metabolize carbohydrates to produce mainly lactate and acetate. These two acids maintain gut microbiota homeostasis and inhibit pathogen overgrowth (53, 56). Intestinal homeostasis reduces the risk of bowel inflammation and improves the human immune system, thus inhibiting tumorigenesis (15, 57). In cell line studies, *Bifidobacterium* species have been observed to inhibit colon cancer cell growth and proliferation (56, 58). However, in an animal study, *Bifidobacterium* species was observed both in normal and colon cancer mice (59). *Lactobacillus* species are common microorganisms that provide various health benefits, including exerting immunomodulatory, antidiabetic, and tumor suppressive effects (54). Some *Lactobacillus* species inhibit CRC tumorigenesis or enhance CRC treatment outcomes (55, 60). However, *Lactobacillus* is a complicated genus containing more than 170 species that can cause opportunistic infections under specific conditions (61, 62).

In the present study, *F. nucleatum* exhibited significantly greater fold changes in abundance in both the PD group and bevacizumab-PD subgroup than in the opposing group and subgroup. *F. nucleatum* is a gram-negative anaerobic bacterium that is strongly associated with CRC initiation and progression (63, 64); mechanisms underlying this association include metabolism alteration, immune modulation, and virulence factor expression (63). *F*. *nucleatum* is more abundant in colorectal adenomas and adenocarcinomas than in the normal colon tissue, and it can promote CRC tumorigenesis (63, 64). Moreover, animal studies have demonstrated that *F*. *nucleatum* can facilitate CRC distant metastasis and intensify chemoresistance (22, 65). Thus, the greater fold change in the abundance of *F*. *nucleatum* in the PD group and the bevacizumab-PD subgroup was expected.

The heterogeneity of our results indicates that interactions among the mCRC, host gene type, chemotherapy and targeted therapy, and gut microbiota involve a complex network instead of a simple pathway. Other studies examining the association between the gut microbiota and CRC tumorigenesis have reported heterogenous results. Liu et al. revealed that the abundance of some CRC-associated bacterial species, such as those of the *Fusobacterium*, *Bacteroides*, and *Prevotella* genera, varied among different biopsy specimens and that the compositions of the gut microbiota significantly differed between the adenoma and adenocarcinoma tissue (41). The composition of the gut microbiota also differed between the proximal and distal CRC tissues. However, the finding of the bacterial analysis performed using stool samples is not equivalent to that conducted using CRC tissues (66). We provide preliminary data regarding the association of the gut microbiota and combined chemotherapy and targeted therapy in mCRC.

*P. dentalis* and *P. copri* exhibited significantly greater fold changes in abundance in both the PR and bevacizumab-PR subgroups than in the PD group and bevacizumab-PD subgroup. *Prevotella* species are associated with the development of CRC because they were discovered to be more abundant in patients with CRC and in the adenoma tissue than in healthy individuals (41, 66, 67). The heterogeneity of *Prevotella* species in adenoma–adenocarcinoma sequences was also noted. Liu et al. reported that the abundance of *Prevotella* species was high in the adenoma tissue but low in the CRC tissue (41). Niccolai et al. demonstrated that *Prevotella* species are positively correlated with interleukin-9. Although interleukin-9 is associated with CRC tumorigenesis, the authors could not confirm the role of *Prevotella* species in CRC development (68). Because all the stool samples in our study were collected from the patients with CRC, a higher abundance of *Prevotella* species in the PR group is expected. However, the effects of *Prevotella* species on CRC treatment, especially with targeted therapy, are uncertain and require further investigation.

The fold change in the abundance of *K*. *quasipneumoniae* in the PR group was nearly 1000 (2^10^) times greater than that of *K*. *quasipneumoniae* in the PD group. *K*. *quasipneumoniae* is closely related to *Klebsiella pneumoniae* (69), and interspecies and intraspecies gene transmission is possible between the two species (70). *K*. *pneumoniae* is a gram-negative pathogen that cause various infectious diseases (71) and is also associated with CRC development because it produces colibactin (72, 73). Colibactin, which was initially reported in *Escherichia coli*, is a bacterial toxin that contributes to CRC tumorigenesis (72–74). *E. coli* carries the polyketide synthase (*pks*) gene that can produce colibactin and induce CRC development (72, 74). *K. pneumoniae* also carry the *pks* gene and produce colibactin, causing DNA damage and subsequent CRC carcinogenesis (72, 73). Lai et al. reported that the incidence rate of *pks*-positive *K. pneumoniae* in Taiwan is approximately 25.6%, which is much higher than the 3.5% rate in Europe (73). Therefore, studying the role of *pks*-positive *K. pneumoniae* in Taiwanese patients undergoing treatment for mCRC is crucial to determine the role of *K. pneumoniae* as a potential target bacterium for future treatments.

Several blind spots were present in our data. For example, *Veillonella* species (*V*. *atypica*, *V*. *dispar*, *and V*. *nakazawae*) were highly abundant in the PR group, but little information is available regarding their clinical effects on CRC. Some bacteria that have been demonstrated to contribute to CRC development, such as *Enterococcus faecalis*, *Bacteroides fragilis*, and *E. coli* (15, 18, 39), were not found to be highly abundant in our results. *Lactobacillus* and *Bifidobacterium* species were highly abundant in the nonfavorable outcome group. Detailed dietary habit history, especially that related to probiotic intake, may be necessary to yield more robust results.

The major limitation of this study is the insufficiently small sample sizes of patients and stool samples. According to the RECIST criteria, the patients with a SD were considered to not exhibit the best response; thus, we excluded these patients from our study (28). Although some studies have classified patients without a PD as responders and compared outcomes between PD and PR + SD groups (75, 76), we identified the bacterial species that have the highest potential to affect mCRC treatment outcomes. Thus, we compared the gut microbiota between the PD and PR groups. This study design resulted in the small size of our patient sample. For instance, anti-EGFR agents are more effective in inducing CRC cytoreduction than do anti-VEGF agents in patients with the wild-type *KRAS* gene and mCRC, which means fewer patients exhibit a PD after using anti-EGFR agents as the first-line treatment (77, 78). In our study, only six patients met the criteria for inclusion in the cetuximab-PD subgroup, which greatly hampered the interpretation of our results. Nevertheless, this pilot study represents the first step to discovering the interaction between the human gut microbiota and targeted therapies for CRC. A larger sample size and more stool samples are required to clarify the role of the gut microbiota in mCRC treatment, especially its interaction with combined chemotherapy and targeted therapy.

## Conclusion

Our results indicate that a higher gut microbiota diversity is associated with a more favorable treatment outcome in patients with mCRC. Considerable heterogeneity was observed in the bacterial species in the stool samples of the patients with mCRC. *Lactobacillus* and *Bifidobacterium* species exhibited significantly greater fold changes in abundance in the PD group than in the PR group. *F. nucleatum* exhibited significantly greater fold changes in abundance in the PD group and the bevacizumab-PD subgroup. *K. quasipneumoniae* exhibited the greatest fold change in abundance among all bacterial species in the PD group. This result warrants further investigation especially in a Taiwanese population. A prospective, randomized study with a larger sample size and greater number of stool samples is necessary to validate the correlation between microbiota and targeted therapy in mCRC.

## Data availability statement

The data presented in the study is deposited in the European Bioinformatics Institute (EMBL-EBI) repository. Accession number is PRJEB55366. The direct link is https://www.ebi.ac.uk/ena/browser/view/PRJEB55366.

## Ethics statement

The studies involving human participants were reviewed and approved by Institutional Ethics Committee of Kaohsiung Medical University Chung-Ho Memorial Hospital. The patients/participants provided their written informed consent to participate in this study.

## Author contributions

Conceptualization, L-HL, D-CW, T-LC, C-YL, and J-YW; Methodology, C-HC, Z-FM, K-LY, C-JL, and C-YL; Software, C-HC and C-YL; Validation, C-YL and J-YW; Visualization, Y-CC and C-HC; Writing—original draft preparation, Y-CC; Writing—review and editing, Y-CC, C-HC, C-YL, and J-YW; All authors contributed to the article and approved the submitted version.

## Funding

This work was supported by grants through funding from the Ministry of Science and Technology (MOST 109-2314-B-037-035, MOST 109-2314-B-037-040, MOST 109-2314-B-037-046-MY3, MOST110-2314-B-037-097) and the Ministry of Health and Welfare (MOHW109-TDU-B-212-134026, MOHW109-TDU-B-212-114006, MOHW110-TDU-B-212-1140026) and funded by the health and welfare surcharge of on tobacco products, and the Kaohsiung Medical University Hospital (KMUH110-0R37, KMUH110-0R38, KMUH110-0M34, KMUH110-0M35, KMUH110-0M36, KMUHSA11013, KMUH-DK(C)110010, KMUH-DK(B)110004-3) and KMU Center for Cancer Research (KMU-TC111A04-1) and KMU Center for Liquid Biopsy and Cohort Research Center Grant (KMU-TC109B05) and KMU Office for Industry-Academic Collaboration (S109036),Kaohsiung Medical University. In addition, this study was supported by the Grant of Taiwan Precision Medicine Initiative and Taiwan Biobank, Academia Sinica, Taiwan, R.O.C.

## Acknowledgments

We acknowledge the support of Kaohsiung Medical University Hospital, Kaohsiung Medical University and Taiwan Academia Sinica.

## Conflict of interest

The authors declare that the research was conducted in the absence of any commercial or financial relationships that could be construed as a potential conflict of interest.

## Publisher’s note

All claims expressed in this article are solely those of the authors and do not necessarily represent those of their affiliated organizations, or those of the publisher, the editors and the reviewers. Any product that may be evaluated in this article, or claim that may be made by its manufacturer, is not guaranteed or endorsed by the publisher.
